# Supplementary material for: Risk factors for sacrococcygeal pilonidal sinus: a systematic review and meta-analysis supplemented by genetic causal assessment
Source: Front Surg. 2026 Jan 7;12:1718589. doi: 10.3389/fsurg.2025.1718589 (PMC12819706; doi:10.3389/fsurg.2025.1718589)
Supplement: Supplementary file 2 [file Datasheet2.zip › Supplementary Data 2/MR_pipeline_p5e-8/leaveone_plots/ukb-saige-272.1.finngen_R12_L12_PILONIDALCYST.pdf]

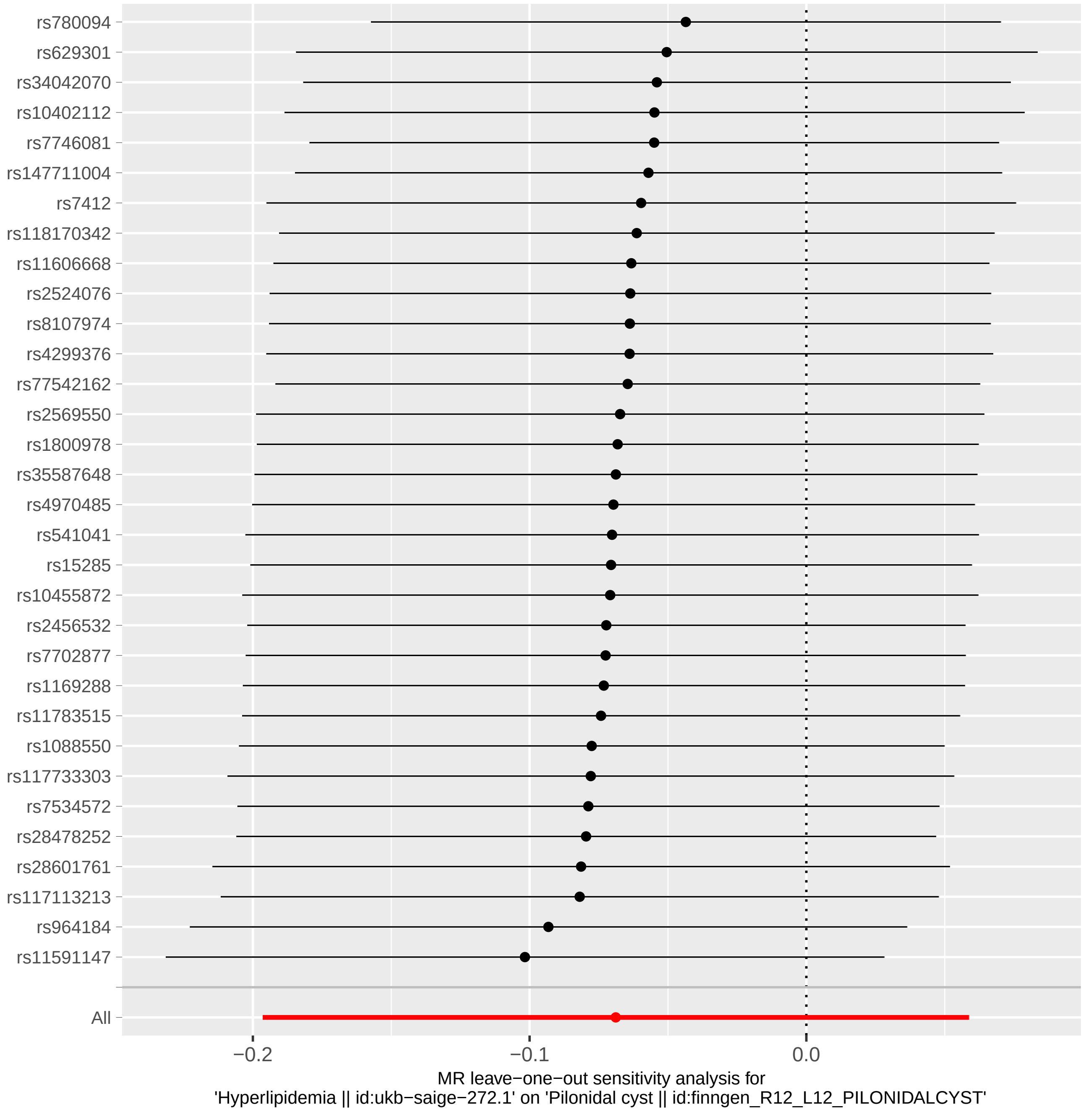

rs780094
rs629301
rs34042070
rs10402112
rs7746081
rs147711004
rs7412
rs118170342
rs11606668
rs2524076
rs8107974
rs4299376
rs77542162
rs2569550
rs1800978
rs35587648
rs4970485
rs541041
rs15285
rs10455872
rs2456532
rs7702877
rs1169288
rs11783515
rs1088550
rs117733303
rs7534572
rs28478252
rs28601761
rs117113213
rs964184
rs11591147
All
−0.2
−0.1
0.0
MR leave−one−out sensitivity analysis for
'Hyperlipidemia || id:ukb−saige−272.1' on 'Pilonidal cyst || id:finngen_R12_L12_PILONIDALCYST'
